# AICAR Induces Apoptosis and Inhibits Migration and Invasion in Prostate Cancer Cells Through an AMPK/mTOR-Dependent Pathway

**DOI:** 10.3390/ijms20071647

**Published:** 2019-04-03

**Authors:** Chia-Cheng Su, Kun-Lin Hsieh, Po-Len Liu, Hsin-Chih Yeh, Shu-Pin Huang, Shih-Hua Fang, Wei-Chung Cheng, Kuan-Hua Huang, Fang-Yen Chiu, I-Ling Lin, Ming-Yii Huang, Chia-Yang Li

**Affiliations:** 1 Division of Urology, Department of Surgery, Chi-Mei Medical Center, Tainan 71004, Taiwan; s2341438@yahoo.com.tw (C.-C.S.); samlin.hsieh@gmail.com (K.-L.H.); skhsteven@gmail.com (K.-H.H.); 2 Graduate Institute of Medicine, College of Medicine, Kaohsiung Medical University, Kaohsiung 80708, Taiwan; fangyen0210@hotmail.com; 3 Department of Senior Citizen Service Management, Chia Nan University of Pharmacy and Science, Tainan 71710, Taiwan; 4 Department of Respiratory Therapy, College of Medicine, Kaohsiung Medical University, Kaohsiung 80708, Taiwan; kisa@kmu.edu.tw; 5 Department of Urology, School of Medicine, College of Medicine, Kaohsiung Medical University, Kaohsiung 80708, Taiwan; patrick1201.tw@yahoo.com.tw (H.-C.Y.); shpihu73@gmail.com (S.-P.H.); 6 Department of Urology, Kaohsiung Municipal Ta-Tung Hospital, Kaohsiung 80145, Taiwan; 7 Department of Urology, Kaohsiung Medical University Hospital, Kaohsiung Medical University, Kaohsiung 80708, Taiwan; 8 Institute of Athletics, National Taiwan University of Sport, Taichung 40404, Taiwan; shfang@ntupes.edu.tw; 9 Graduate Institute of Biomedical Sciences, and Research Center for Tumor Medical Science, China Medical University, Taichung 40402, Taiwan; cwc0702@gmail.com; 10 Department of Medical Laboratory Science and Biotechnology, College of Health Sciences, Kaohsiung Medical University, Kaohsiung 80708, Taiwan; linili@kmu.edu.tw; 11 Department of Radiation Oncology, Kaohsiung Medical University Hospital, Kaohsiung 80756, Taiwan; 12 Department of Radiation Oncology, College of Medicine, Kaohsiung Medical University, Kaohsiung 80708, Taiwan; 13 Center for Biomarkers and Biotech Drugs, Kaohsiung Medical University, Kaohsiung 80708, Taiwan; 14 Center for Infectious Disease and Cancer Research, Kaohsiung Medical University, Kaohsiung 80708, Taiwan

**Keywords:** AICAR, AMPK, prostate cancer, metastasis, chemosensitivity

## Abstract

Current clinical challenges of prostate cancer management are to restrict tumor growth and prohibit metastasis. AICAR (5-aminoimidazole-4-carbox-amide-1-β-d-ribofuranoside), an AMP-activated protein kinase (AMPK) agonist, has demonstrated antitumor activities for several types of cancers. However, the activity of AICAR on the cell growth and metastasis of prostate cancer has not been extensively studied. Herein we examine the effects of AICAR on the cell growth and metastasis of prostate cancer cells. Cell growth was performed by MTT assay and soft agar assay; cell apoptosis was examined by Annexin V/propidium iodide (PI) staining and poly ADP ribose polymerase (PARP) cleavage western blot, while cell migration and invasion were evaluated by wound-healing assay and transwell assay respectively. Epithelial–mesenchymal transition (EMT)-related protein expression and AMPK/mTOR-dependent signaling axis were analyzed by western blot. In addition, we also tested the effect of AICAR on the chemosensitivity to docetaxel using MTT assay. Our results indicated that AICAR inhibits cell growth in prostate cancer cells, but not in non-cancerous prostate cells. In addition, our results demonstrated that AICAR induces apoptosis, attenuates transforming growth factor (TGF)-β-induced cell migration, invasion and EMT-related protein expression, and enhances the chemosensitivity to docetaxel in prostate cancer cells through regulating the AMPK/mTOR-dependent pathway. These findings support AICAR as a potential therapeutic agent for the treatment of prostate cancer.

## 1. Introduction

Prostate cancer is the most common cancer and the second leading cause of cancer-related death among men in the United States [1]. Current treatment options for prostate cancer include surgery, hormonal therapy, chemotherapy, radiation therapy, radiofrequency ablation, high-intensity focused ultrasound, cryotherapy, and cancer vaccine [2,3]. Androgen deprivation therapy (ADT) by surgical or chemical castration has been the mainstay of treatment for advanced prostate cancer in the past few decades. However, the majority of androgen-sensitive prostate cancer patients will eventually develop resistance to ADT within 1 to 3 years and the disease will become androgen-independent [4]. Docetaxel chemotherapy was administered to patients with metastatic castration-resistant prostate cancer (CRPC) for a decade, and demonstrated ability to improve the median overall survival by around 3 months [5,6]. Currently, there is no effective therapy for recurrent prostate cancer; hence, a novel therapeutic method for CRPC is needed.

CRPC is often chartered by the reactivation of the androgen receptor (AR) transcriptional activity with castrate levels of androgens because of amplification of AR gene and AR mutation [7,8]. Although AR is the classical target for prostate cancer prevention and treatment, several recent studies indicated that estrogen and estrogen receptor (ER) have also been implicated in prostate cancer development, prostate cancer cell stemness, and tumor progression [8,9]. There are two classes of ER, ERα, and ERβ, which exhibit opposite roles in the progression of prostate cancer. ERα is considered as an oncogene, which mediates the detrimental effects of estrogen including proliferation, inflammation, and carcinogenesis [8], whereas, ERβ acts as an anti-oncogenic role through protecting prostatic transformation, preventing proliferation and regulating DNA methylation [8]. Therefore, targeting ER might be an effectively strategy to limit the growth and spread of prostate cancer.

The 5′-adenosine monophosphate (AMP)-activated protein kinase (AMPK) is activated by increases in cellular ATP/AMP ratio and plays an important role in regulating glycolytic activity and maintaining energy balance at both cellular and whole body levels [10]. Previous studies indicated that AMPK exhibits intricate relations with other energy/metabolite sensor pathways (e.g., SIRT1, Akt, mTOR, and PARPs) and acts in a coordinated fashion with these [11,12,13,14,15]. AMPK induction leads to enhanced mitochondrial oxidation and mitochondrial biogenesis that have been shown to exert anti-Warburg and anti-proliferative effects in several types of cancers, such as leukemia [16], breast cancer [17], pancreatic cancer [18], hepatocellular carcinoma [19], and prostate cancer [20]. These findings suggest that AMPK activation might be used beneficially for cancer treatment.

The adenoside analog compound, 5-aminoimidazole-4-carbox-amide-1-β-d-ribofuranoside (AICAR), is intracellularly converted by adenosine kinase to the non-phosphorylated derivative amino-imidazolecarboxamide ribonucleotide (ZMP), an analogue of AMP, which activates AMPK [21]. Therefore, AICAR is often used as an activator of AMPK [22] to modulate cellular energy homeostasis, although AMPK-independent effects have also been proposed [23,24]. A previous study indicated that AICAR inhibited the growth of androgen-independent (DU145, PC3) and androgen-sensitive (LNCaP) cells after four days of treatment [25]. In addition, AICAR inhibited two key enzymes involved in protein synthesis, mTOR and p70S6K, and blocked the ability of the androgen R1881 (methyltrienolone; a synthetic androgen agonist) to increase cell growth and the expression of two enzymes for de novo fatty acid synthesis, acetyl CoA carboxylase and fatty acid synthase, in the LNCaP cells [25]. A current study showed that AICAR induced AMPK-independent programmed necrosis in prostate cancer cells [26]. 

Collectively, these studies indicate that AICAR has potential in inhibiting the growth of prostate cancer. However, the effect of AICAR on the apoptosis and migration of prostate cancer remains unclear. In the present study, we evaluated the effects of AICAR on apoptotic activity, migration, invasion and chemosensitivity through AMPK phosphorylation in human prostate cancer cells. 

## 2. Results

### 2.1. AICAR Inhibits Cell Growth in Prostate Cancer Cells, but Not in Non-Cancerous Prostate Cells

**Figure 1:**
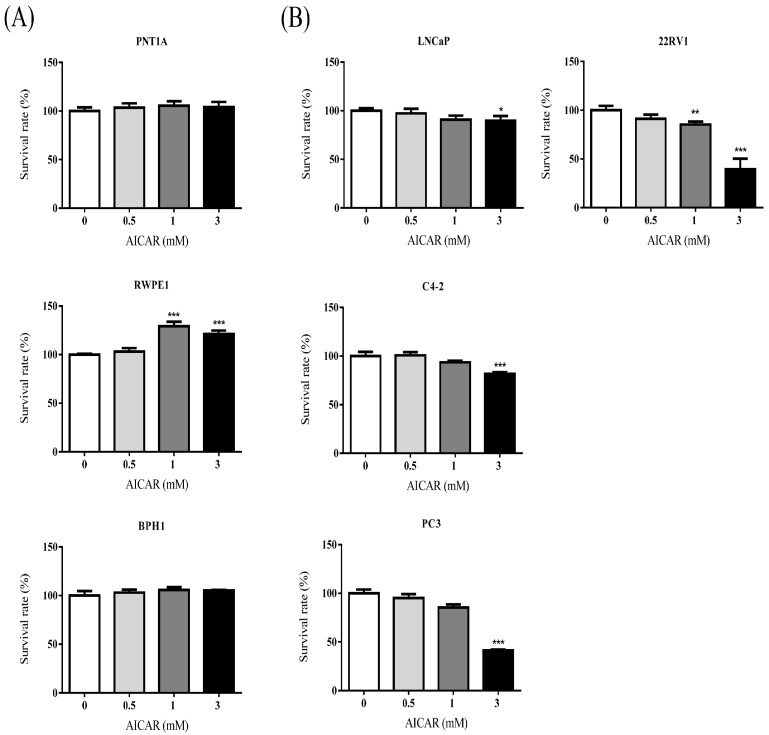
The effect of AICAR (5-aminoimidazole-4-carbox-amide-1-β-d-ribofuranoside) on the growth of non-cancerous or cancerous prostate cells. (**A**) non-cancerous or (**B**) cancerous prostate cells were treated with different concentrations of AICAR for 24 h. The cell viability was measured by the MTT assay. Data are represented as means ± SD of triplicate values and statistical significance was determined using the Student’s t-test (* *p* < 0.05; ** *p* < 0.01; *** *p* < 0.001).

**Figure 2:**
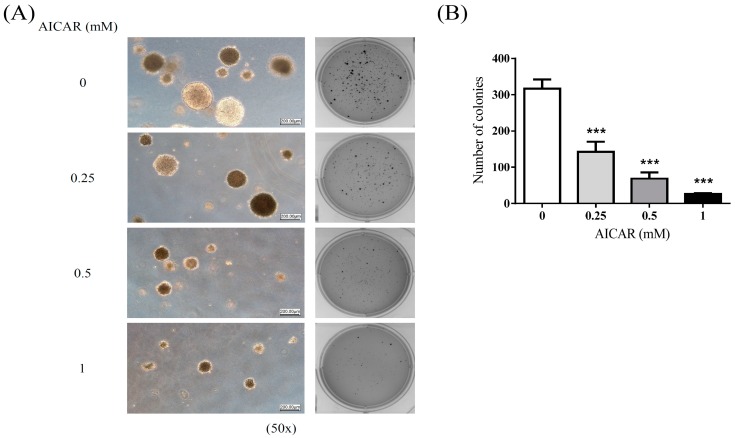
The effect of AICAR on growth under anchorage-independent conditions of prostate cancer cells. 22Rv1 cells were treated with different concentrations of AICAR, and then grown in soft agar, an anchorage-independent condition, for 3 weeks. (**A**) Colonies were stained with crystal violet and captured using the Bio-Rad ChemiDoc XRS+ system (Hercules, CA, USA). (**B**) Data are quantified and represented as means ± SD of triplicate values and statistical significance was determined using the Student’s t-test (*** *p* < 0.001).

To examine the effect of AICAR on the growth of non-cancerous or cancerous prostate cells, cells were treated with various concentrations (0, 0.5, 1, and 3 mM) of AICAR for 24 h. Cell viability was analyzed by MTT assay. The experimental results showed that AICAR did not inhibit the growth of non-cancerous prostate cells, PNT1A and BPH1 cells, as well as having pro-survival effect in RWPE-1 cells (Figure 1A), although we found that AICAR had inhibitory effects on the cell growth in cancerous prostate cells, especially in 22Rv1 and PC3 cells (Figure 1B). A previous study also showed that AICAR inhibits the growth of androgen-independent (DU145, PC3) and androgen-sensitive (LNCaP) cells [25]; however, the effect of AICAR on 22Rv1 cells has not been examined previously. The following experiments used 22Rv1 cells to explore the inhibitory effect of AICAR in prostate cancer. To examine whether AICAR affects the colony growth under anchorage-independent conditions, 22Rv1 cells were treated with different concentrations (0, 0.25, 0.5 and 1 mM) of AICAR and then a soft agar assay was conducted. The experimental results showed that AICAR suppressed growth of 22Rv1 cells in soft agar in a dose-dependent manner (Figure 2).

### 2.2. AICAR Induces Apoptosis in Prostate Cancer Cells

**Figure 3:**
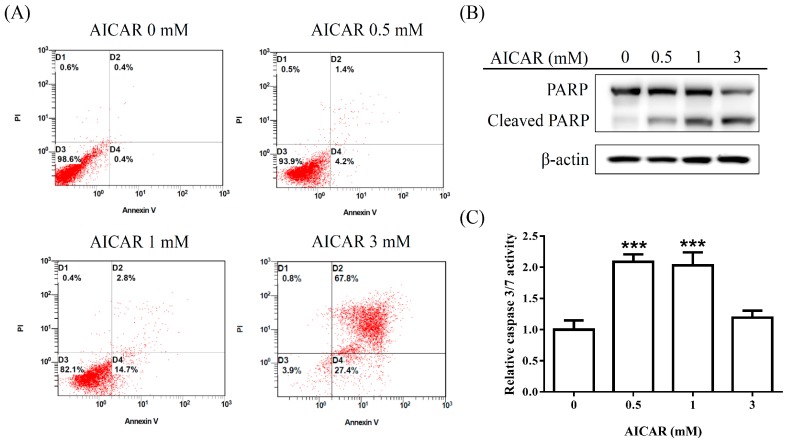
Effect of AICAR on the apoptosis in 22Rv1 prostate cancer cells. Cells were incubated with different concentrations of AICAR for 24 h. (**A**) Cells were collected, stained with Annexin V and propidium iodide (PI), and analyzed by flow cytometry. Data are representative of at least three independent experiments with similar results. (**B**) The expression of Poly(ADP-ribose) polymerase (PARP) was determined by western blot. Actin was used as a loading control in western blot. (**C**) Cellular caspase 3/7 activities were analyzed with caspase-glo assay kit. Data are represented as means ± SD of triplicate values and statistical significance was determined using the Student’s t-test (*** *p* < 0.001).

To further test whether AICAR induces apoptosis in prostate cancer cells, 22Rv1 cells were treated with various concentrations (0, 0.5, 1, and 3 mM) of AICAR for 24 h. Apoptotic cells were detected with Annexin V/PI staining using flow cytometry. Our results demonstrated that AICAR induced apoptosis (Figure 3A). Poly(ADP-ribose) polymerase (PARP) is a nuclear enzyme involved in DNA repair, which is cleaved by caspase-3 during apoptosis [27]. We further examined whether AICAR affects the expression of PARP in 22Rv1 cells. As shown in Figure 3B, AICAR increased the expression of cleaved PARP, an apoptosis marker, in 22Rv1 cells. In addition, we also examined the activity of caspase 3/7 using a luminescent substrate-based assay. Our results indicated that AICAR increased the activity of caspase 3/7 in 22Rv1 cells (Figure 3C).

### 2.3. AICAR Inhibits Transforming Growth Factor-β (TGF-β)-Induced Epithelial to Mesenchymal Transition (EMT) and Attenuates TGF-β-Induced Migration and Invasion Activities 

**Figure 4:**
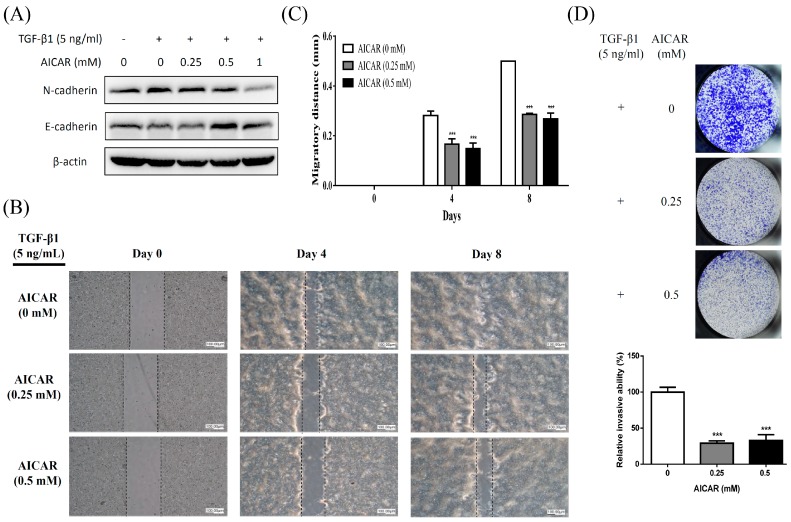
Effect of AICAR on transforming growth factor-β (TGF)-β-induced epithelial to mesenchymal transition (EMT), migration, and invasion in 22Rv1 prostate cancer cells. (**A**) Cells were treated with 5 ng/mL TGF-β1 and different concentrations of AICAR for 72 h. The expression of N-cadherin and E-cadherin was analyzed by western blot. Actin was used as a loading control in western blot. The western blotting results are representative of results obtained in three separate experiments. (**B**) Cells were seeded in SPLScarTM Block overnight, the block was then removed, and cells were treated with 5 ng/mL TGF-β1 and different concentrations of AICAR. Cell migration was monitored under a phase-contrast microscope. Data are representative of at least three independent experiments with similar results. (**C**) The migratory distance was calculated. (**D**) Cells were treated with 5 ng/mL TGF-β1 and different concentrations of AICAR, then seeded into Matrigel transwell inserts for 5 days. The invaded cells were quantified using crystal violet staining. Data are represented as means ± SD of triplicate values and statistical significance was determined using the Student’s *t*-test (* *p* < 0.05; ** *p* < 0. 01; *** *p* < 0.001).

TGF-β signaling is well known as a key regulator of many biological processes in prostate cancer including inducing EMT, migration and metastasis [28]. To examine whether AICAR affects TGF-β-induced EMT, migration, and invasion activities in prostate cancer cells, 22Rv1 cells were treated with 5 ng/mL TGF-β and various concentrations (0, 0.25, and 0.5 mM) of AICAR. The expression of EMT-related proteins was analyzed using western blot. As shown in Figure 4A, AICAR inhibited TGF-β-induced EMT through inhibiting the expression of mesenchymal marker, N-cadherin, and enhancing the expression of epithelial marker, E-cadherin. Cell migration and invasion were performed by wound-healing assay and Matrigel transwell assay respectively. Our results showed that AICAR significantly inhibited TGF-β-induced migration (Figure 4B,C) and invasion (Figure 4D). 

### 2.4. AICAR Exhibits Synergistic Effect with Docetaxel Treatment 

**Figure 5:**
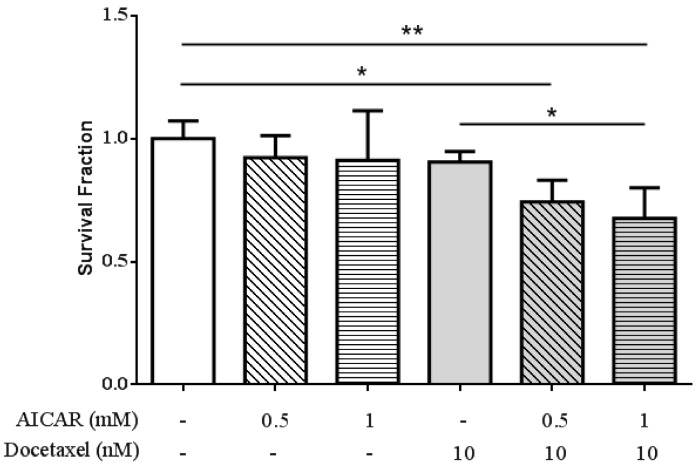
Effect of AICAR on the sensitivity of 22Rv1 prostate cancer cells to docetaxel treatment. Cells were treated with different concentrations of AICAR in the presence or absence of docetaxel for 24 h. The cell viability was measured by the MTT assay. Data are represented as means ± SD of triplicate values and statistical significance was determined using the Student’s *t*-test (* *p* < 0.05; ** *p* < 0.01).

To examine whether AICAR affects the therapeutic efficiency of docetaxel-based therapy in prostate cancer, 22Rv1 cells were treated with various doses (0, 0.5 and 1 mM) of AICAR in the presence or absence of docetaxel for 24 h. Cell survival was analyzed by MTT assay. As shown in Figure 5, AICAR exhibited synergistic effect with docetaxel treatment in prostate cancer cells.

### 2.5. AICAR Inhibits the Growth of Prostate Cancer Cells Through Activating an Ampk/Mtor-Dependent Pathway

**Figure 6:**
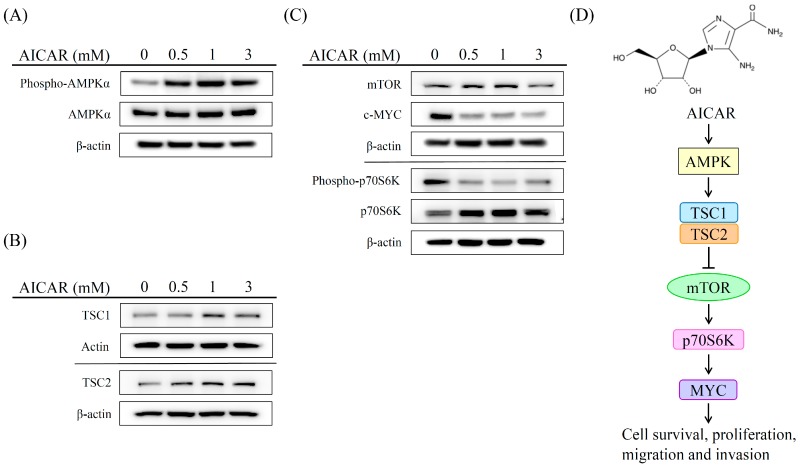
Effect of AICAR on the AMPK/mTOR-dependent pathway in 22Rv1 prostate cancer cells. Cells were treated with different concentrations of AICAR for 2 h. The expression of (**A**) phospho-AMPK, AMPK, (**B**) TSC1 and TSC2 was examined by western blot. Cells were treated with different concentrations of AICAR for 6 h. (**C**) The expression of mTOR, cMYC, phosphor-p70S6K and p70S6K was determined by western blot. Actin was used as a loading control in western blot. The western blotting results are representative of results obtained in three separate experiments. (**D**) Proposed mechanism of the anticancer effects induced by AICAR in 22Rv1 prostate cancer cells.

AMPK is an upstream regulator in modulating the activation of TSC1 and TSC2 which consequently inhibits cell survival and proliferation via repressed mTOR-p70S6K-MYC signaling pathway [29,30]. To investigate the mechanism of action of AICAR in regulating the activity of the AMPK-dependent pathway, 22Rv1 cells were treated with different concentrations (0, 0.5, 1, and 3 mM) of AICAR. The expression of phospho-AMPK, AMPK, TSC-1, TSC-2, mTOR, phospho-p70S6K, p70S6K, and MYC was analyzed by western blot. As shown in Figure 6A, AICAR promoted the phosphorylation of AMPK in 22Rv1 cells. In addition, our experimental results showed that AICAR enhanced the expression of TSC1 and TSC2 (Figure 6B), whereas AICAR reduced the expression of mTOR and MYC as well as decreased the phosphorylation of p70S6K (Figure 6C). These results suggest that AICAR inhibits the growth of prostate cancer cells through an AMPK/mTOR-dependent pathway (Figure 6D). 

## 3. Discussion

AMPK is an important regulator of cellular energy homeostasis [31]. Several agents have been demonstrated to activate AMPK, including metformin and phenformin, which increase the AMP:ATP ratio, the nucleoside AICAR which is metabolized to an AMP mimetic, and A769662, which is a direct activator of AMPK [32]. Although a number of studies have indicated an anti-cancer role for AMPK [14,16,17,19,20,33,34], the effect of an AMPK activator, AICAR, in regulating the pathological processes of prostate cancer, remains enigmatic. Previous studies have shown that AICAR could exert cytotoxic effect on cancer cells via AMPK-dependent and/or MPK-independent mechanisms [21,33,35,36]; however, the cytotoxic effect on prostate cancer remains controversial. Guo et al. indicated that AICAR induces programmed necrosis, but not apoptosis, in prostate cancer cells, LNCaP, PC-3, and PC-82 cells through AMPK-independent pathways [26]. In contrast, Sauer et al. demonstrated that AICAR induces apoptosis of DU-145 prostate cancer cells through the AMPK/mTOR-dependent signaling pathway [21]. Our experimental results indicated that AICAR inhibits the growth of cancerous prostate cells, but not non-cancerous prostate cells. Furthermore, we found that AICAR induces apoptosis, but not necrosis, in 22Rv1 prostate cancer cells. These results point out that AICAR inhibits cell growth and induces cell death in different prostate cancer cell lines probably through different mechanisms. In addition, AMPK is an upstream regulator in modulating the activation of TSC1 and TSC2, which consequently inhibits cell survival and proliferation via repressed mTOR-p70S6K-MYC signaling pathway [29,30]. Our experimental results showed that AICAR enhances the expression of TSC1 and TSC2, whereas it reduces the expression of mTOR and MYC as well as decreases the phosphorylation of p70S6K in 22Rv1 prostate cancer cells. We suggest that AICAR induces apoptosis and inhibits migration of prostate cancer cells through an AMPK/mTOR-dependent pathway.

Cancer metastasis is the major cause of morbidity and mortality; approximately 80% of the men who have expired from prostate cancer possessed bone metastases [37]. Our experimental results indicated that AICAR suppresses TGF-β-induced cell migration and invasion as well as inhibits TGF-β-induced EMT by decreasing the expression of a mesenchymal-related marker, N-cadherin and enhancing the expression of an epithelial-related marker, E-cadherin in 22Rv1 prostate cancer cells. A recent study also indicated that treatment of AICAR suppresses migration and invasion in PC3 and PC3M prostate cancer cells [20]. These results imply that AICAR might have potential in inhibiting the metastatic activity in prostate cancer.

Previous studies have indicated that ER mediated signaling axis controls prostate cancer growth and progression. Ma et al. indicated that metformin enhances tamoxifen-mediated tumor growth inhibition in ER-positive breast carcinoma [38]. Zhang et al. demonstrated that Metformin significantly decreased E2-stimulated cell proliferation; an effect that was rescued in the presence of compound C (an AMPK inhibitor) [39]. Moreover, metformin significantly inhibited ERα expression while increasing ERβ expression, whereas treatment with compound C reversed these effects [39]. These results suggest that the activation of AMPK is associated with ER-mediated signaling axis. Our results showed that AICAR has more inhibitory effect in CRPC cells (22Rv1 and PC3) than androgen-dependent prostate cancer cells (LNCaP and C4-2), suggesting that inhibitory effect of AICAR might be associated with the activity of the ER-mediated signaling axis. 

Docetaxel is a first-line treatment for CRPC, which confers survival advantages of approximately 2 months for patients with low overall survival benefit [40]; however, treatment with docetaxel usually causes adverse side effects including hair loss, myelosuppression, neurotoxicity, and diarrhea. Our experimental results showed that AICAR has synergistic effect with docetaxel treatment. These results suggest that AICAR increases the sensitization of prostate cancer cells to docetaxel-induced apoptosis, which might have benefit for reducing toxicity of chemotherapy in prostate cancer patients.

In summary, our experimental results indicated that AICAR inhibits cell growth, induces apoptosis, attenuates cell migration, enhances chemosensitivity to docetaxel, and suppresses the activation of the AMPK/mTOR-dependent signaling pathway. These results suggest that AICAR appears as a new potential anticancer agent for treating prostate cancer.

## 4. Materials and Methods 

### 4.1. Reagents

RPMI 1640 medium, F-12 medium, penicillin, streptomycin and fetal bovine serum (FBS) were purchased from Gibco-BRL (Life Technologies, Grand Island, NY, USA). AICAR, bovine serum albumin (BSA), phosphate-buffered saline (PBS), RIPA buffer, protease inhibitor cocktail, phosphatase inhibitor cocktail, stripping buffer, thioglycollate medium, and 3-(4,5-dimethylthiazol- 2-yl)-2, 5-diphenyl tetrazolium bromide (MTT) were purchased from Sigma Aldrich (St. Louis, MO, USA). AICAR was purchased from Cayman Chemical (Ann Arbor, MI, USA). Alexa Fluor® 488 Annexin V/Dead Cell Apoptosis Kit was purchased from Thermo Fisher Scientific (Waltham, MA, USA). BCA protein assay reagent was purchased from Thermo Scientific. For western blotting, rabbit antibodies against human phospho-AMPK, AMPK, MYC, mTOR, PARP, phospho-p70S6K, p70S6K, TSC-1, TSC-2, β-actin and secondary antibodies were purchased from Cell Signaling (Farmingdale, NY, USA). Mouse antibodies against human N-cadherin and E-cadherin were purchased from BD Biosciences (San Jose, CA, USA). Caspase-Glo 3/7 assay kit was purchased from Promega (Madison, WI, USA). Transforming growth factor-beta 1 was purchased from PeproTech (Rocky Hill, NJ, USA). Docetaxel (Taxothere, 20 mg/mL) was obtained from Sanofi Aventis (Berlin, Germany).

### 4.2. Cell Culture

Human normal prostate epithelial cell lines (PNT1A and RWPE1), human benign prostatic hyperplasia epithelial cell line (BPH1), and human prostate cancer cell lines (C4-2 and PC3) were obtained from Professor Jer-Tsong Hsieh (Department of Urology, Southwestern Medical Center, University of Texas, TX, USA). Human prostate cancer cell lines (LNCaP and 22Rv1) were purchased from Bioresource Collection and Research Center (Food Industry Research and Development Institute, Hsinchu, Taiwan). Cells were cultured in RPMI 1640 medium (PNT1A, RWPE1, BPH1, LNCaP, C4-2, and 22Rv1) or F-12 medium (PC3), supplemented with antibiotics (100 U/mL penicillin and 100 U/mL streptomycin) and 10% (*v*/*v*) FBS in a humidified atmosphere of 5% CO_2_ at 37 °C and passaged every 2–3 days to maintain growth.

### 4.3. MTT Assay 

Cells were seeded in a 96-well plate at a concentration of 1 × 10^5^ cells, and were allowed to acclimatize overnight. Cells were treated with various concentrations of the AICAR (0, 0.5, 1, and 3 mM) for 24 h. Cell viability was measured by the ability of viable cells to reduce MTT to formazan based on the ability of living cells utilized thiazolyl blue and converted it into purple formazan. The concentration of formazan is measured by determining the OD at 570 nm using a microplate reader (BioTek Instruments, Inc., Winooski, VT, USA), and the results are given as relative percentage to the untreated control. To detect the synergistic effects of AICAR, cells were treated with different doses of the AICAR (0, 0.5, and 1 mM) combined with different doses of docetaxel for 24 and 48 h. 

### 4.4. Soft Agar Colony Formation Assay

Noble agar (BD Biosciences, Franklin Lakes, NJ, USA) was dissolved in complete medium and coated with 0.5% agar solution on the bottom of 6-well plates. After solidifying, top agar medium mixture (0.3%) containing 5 × 10^3^ cells was added, and incubated at 37 °C in a humidified atmosphere of 5% CO_2_ for 3 weeks. Colonies were stained with 0.05% crystal violet-10% ethanol in PBS. Photographs of the stained colonies were captured using Bio-Rad ChemiDoc XRS^+^ system (Bio-Rad Laboratories, Inc., Hercules, CA, USA) and quantified using ImageJ software (National Institutes of Health, Bethesda, MD, USA).

### 4.5. Apoptosis Assay 

22Rv1 cells were treated with different doses of AICAR (0, 0.5, 1, and 3 mM) for 24 hr. Cells were trypsinized, washed twice by cold PBS, and stained with Alexa Fluor^®^ 488 Annexin V and propidium iodide (PI) according to the manufacturer’s protocol (Thermo Fisher Scientific). Apoptotic cells were determined using FC500 flow cytometer (Beckman-Coulter, Fullerton, CA, USA). Ten thousand events were collected per sample. Data were analyzed by CXP analysis software (Beckman-Coulter, Fullerton, CA, USA).

### 4.6. Western Blot 

Cells were lysed by RIPA buffer with protease inhibitors and phosphatase inhibitors according to the manufacturer’s protocol (Sigma Aldrich, St. Louis, MO, USA). The protein concentration was determined using the BCA protein assay reagent according to the manufacturer’s instructions (Thermo Scientific, Waltham). Cellular protein extracts were separated by electrophoresis using 8% SDS polyacrylamide gel and were electroblotted onto PVDF membranes. The membranes were incubated with blocking solution for 1 hr at room temperature, followed by incubation overnight with primary antibodies at 4 °C (1:1000). Blots were washed three times with Tris-buffered saline/Tween 20 (TBST) and incubated with a 1:5000 dilution of horseradish peroxidase conjugated secondary antibody for 1 h at room temperature. Blots were again washed three times with TBST and developed using an ECL chemiluminescence substrate (Thermo Scientific). The signals were captured and the band intensities were quantified using Bio-Rad ChemiDoc XRS^+^ system (Bio-Rad Laboratories, Inc.).

### 4.7. Caspase 3/7 Activity Assay

22Rv1 cells were seeded in a 96-well white plate at a concentration of 2.5 × 10^4^ cells, and were allowed to acclimatize overnight. Cells were treated with various concentrations of the AICAR (0, 0.5, 1, and 3 mM) for 24 h. A total of 100 μL Caspase-Glo^®^ 3/7 reagent was added to each well, gently mixed contents of wells using a plate shaker at 300~500 rpm for 30 s, and then samples were incubated for 30 min at RT. Enzyme activity is directly proportional to luminescence, which was measured using a luminescence microplate reader (BioTek Instruments, Inc.). Data were normalized relative to the caspase 3/7 activity of cells treated with DMSO alone.

### 4.8. Migration Assay

SPLScarTM Block (SPL life sciences, Korea) was placed in a 24-well plate, seeded 5 × 10^4^ cells in each side of block, and was allowed to acclimatize overnight. The block was removed, the flash medium replaced, and cells were treated with 5 ng/mL TGF-β1 and different concentrations of AICAR. Cell migration was monitored under a phase-contrast microscope and the migratory distance was calculated. 

### 4.9. Invasion Assay

24-well hanging inserts with 8 µm polyester membrane (Millipore Co., Billerica, MA, USA) were used and coated with Matrigel according to the manufacturer’s protocol (BD Biosciences, San Jose, CA, USA). A total of 5 × 10^4^ cells in 200 μL serum-free medium containing indicated dosage of TGF-β1 and AICAR were seeded into the upper chamber and 600 μL complete medium (10% FBS as chemo attractants) containing indicated dosage of TGF-β1 and AICAR was added in the lower chamber. Following incubation for 5 days, non-invading cells were carefully removed with a cotton swab. The cells that passed through the filter were fixed with 5% glutaldehyde and stained by 0.1% crystal violet, then photographed. For the quantification of invasiveness, crystal violet was dissolved by 50% methanol and the absorbance was determined at 570 nm using a microplate reader (BioTek Instruments, Inc.).

### 4.10. Statistical Analysis

All data are expressed as means ± SD. Each value is the mean of three independent experiments. Statistical analysis was assessed via Student’s *t*-test using IBM SPSS Statistics v.19 (IBM Corp., Armonk, NY, USA), and the significant difference was set at * *p* < 0.05; ** *p* < 0.01; *** *p* < 0.001.

## Abbreviations

AICAR	5-aminoimidazole-4-carbox-amide-1-β-d-ribofuranoside
AMPK	AMP-activated protein kinase
ADT	Androgen deprivation therapy
CRPC	Castration-resistant prostate cancer
AR	Androgen receptor
ER	Estrogen receptor
FBS	Fetal bovine serum
BSA	Bovine serum albumin
PBS	Phosphate-buffered saline
MTT	3-(4,5-dimethylthiazol-2-yl)-2,5-diphenyl tetrazolium bromide
PI	Propidium iodide
TBST	Tris-buffered saline/Tween 20
PARP	Poly(ADP-ribose) polymerase
TGF-β	Transforming growth factor-beta
EMT	Epithelial to mesenchymal transition
